# Immune Response after SARS-CoV-2 Vaccination in Kidney Transplant Patients

**DOI:** 10.3390/medicina57121327

**Published:** 2021-12-03

**Authors:** Ruta Vaiciuniene, Brigita Sitkauskiene, Inga Arune Bumblyte, Egle Dalinkeviciene, Edita Ziginskiene, Dovydas Bagdonas, Ruta Augliene, Kristina Petruliene, Irmante Bagdziuniene, Inga Skarupskiene, Asta Stankuviene, Jolanta Sauseriene, Sarunas Macinskas, Leonas Valius

**Affiliations:** 1 Department of Nephrology, Lithuanian University of Health Sciences, LT-50161 Kaunas, Lithuania; ingaarune.bumblyte@lsmuni.lt (I.A.B.); egle.dalinkeviciene@lsmu.lt (E.D.); edita.ziginskiene@lsmuni.lt (E.Z.); ruta.augliene@lsmuni.lt (R.A.); kristina.petruliene@lsmu.lt (K.P.); irmante.stramaityte@lsmu.lt (I.B.); inga.skarupskiene@lsmuni.lt (I.S.); asta.stankuviene@lsmu.lt (A.S.); 2 Department of Immunology and Allergology, Lithuanian University of Health Sciences, LT-50161 Kaunas, Lithuania; brigita.sitkauskiene@lsmuni.lt (B.S.); dovydas.bagdonas@kaunoklinikos.lt (D.B.); 3 Department of Family Medicine, Lithuanian University of Health Sciences, LT-50161 Kaunas, Lithuania; jolanta.sauseriene@lsmu.lt (J.S.); sarunas.macinskas@kaunoklinikos.lt (S.M.); Leonas.Valius@lsmuni.lt (L.V.)

**Keywords:** COVID-19, vaccination, response, kidney transplantation

## Abstract

*Background and Objectives*: The prospective study was conducted to evaluate humoral and cellular immune responses after two doses of BNT162b2 (Pfizer-BioNTech) vaccine and possible relation with other factors (medication, etc.) in kidney transplant patients. *Materials and Methods*: Out of 167 vaccinated patients, 136 agreed to a follow-up visit three to six weeks after vaccination. *Results*: Only 39 patients (29%) developed antibody response against SARS-CoV-2 (≥35.2 binding antibody units (BAU)/mL) after full vaccination. Multivariate binary logistic regression analysis showed that predictive factors for good antibody response to the COVID-19 vaccine were better kidney function, higher hemoglobin level, and no use of mycophenolate mofetil for immunosuppression. For seropositive kidney transplant patients there was a significant negative correlation between anti-SARS-CoV-2 antibody titer and CD4/CD8 ratio (Spearman’s correlation coefficient −0.4, *p* = 0.02), percentage of CD19+ cells (r = −0.37, *p* = 0.02), and a positive correlation with percentage of CD8+ cells (r = 0.4, *p* = 0.01). There was an increase of total leucocyte count after vaccination in the total studied population, and in the group of responders. *Conclusions*: Only one third of kidney transplant patients develop sufficient antibody responses after full COVID-19 vaccination with Pfizer-BioNTech. Better kidney function, higher hemoglobin level, and no use of mycophenolate mofetil for immunosuppression increases the adequacy of response. The antibody titers correlated positively with relative number of CD8+ cells and negatively with CD4/CD8 ratio in responders.

## 1. Introduction

Chronic kidney disease (CKD) patients have been significantly impacted during the coronavirus disease of 2019 (COVID-19) pandemic, resulting in a substantial decrease in transplant activity, an increased risk of COVID-19 disease, hospitalization and mortality [1,2,3,4]. Patients with CKD have higher prevalence of other chronic diseases such as hypertension, diabetes and cardiovascular disease, and these comorbidities increase the risk of severity of COVID-19 infection [5,6]. The mortality rate related to COVID-19 infection was unusually high among kidney transplant patients and reached 20–28% as compared with 1–5% in the general population [7,8,9,10]. Vaccination is the most important way to prevent infection. Nephrology societies around the world have recommended prompt, urgent vaccination of CKD patients [11,12]. Immunocompromised patients such as CKD, dialyzed and kidney transplant recipients are one of the most vulnerable groups in the population, are a priority in terms of receiving the COVID-19 vaccination [13,14]. Different vaccines received emergency use authorization by agencies around the globe. The reported efficacy ranged from 50.4% for an inactivated vaccine candidate to 91.6%, 94.1% and 95% for Gam-COVID-Vac, mRNA-1273 and BN162b2. Exclusion criteria in these vaccine studies included the chronic use of immunosuppressive therapy and thus no data on efficacy in kidney transplant patients were available before vaccination [15]. From the earlier studies we know that CKD patients may have a reduced response to vaccines. Seroconversion induced by the hepatitis B virus (HBV) vaccine in patients with CKD is significantly lower than those in the general population [16,17,18]. Studies in transplant patients with immunosuppression also demonstrates an attenuated response to vaccination [19,20,21].

The BNT162b2 (Pfizer-BioNTech) vaccine was the first available COVID-19 vaccine in Lithuania during this pandemia, and organ transplant recipients had vaccination priority. Kidney transplant recipients followed in our transplant center of the Hospital of the Lithuanian University of Health Sciences were able to receive full vaccination during January–February 2021. The aim of our study was to evaluate humoral and cellular immune responses after two doses of BNT162b2 (Pfizer-BioNTech) vaccine and possible relation with other factors (medication, etc.) in kidney transplant patients.

## 2. Materials and Methods

The prospective study was conducted for evaluation of the response to vaccination against SARS-CoV-2 in patients after renal transplantation in the Hospital of Lithuanian University for Health Sciences, Kauno klinikos. The study was approved by the Regional Bioethical Committee (5 February 2021 Nr. BE-2-43).

Out of 317 patients, followed in our center, 167 were vaccinated with two doses of BNT162b2 (Pfizer-BioNTech) SARS-CoV-2 mRNA vaccine with an interval of 21 days as recommended by the manufacturer. Vaccination was recommended not earlier than three months after kidney transplantation. 136 patients agreed to participate in the study and signed written informed consent, and they came for a follow-up visit three to six weeks after vaccination (mean time 35 ± 11.5 days). None of these subjects had COVID-19 previously. Data about comorbid conditions and medications used were collected from their medical records. Anamnesis of angina pectoris, myocardial infarction, and stroke was counted as cardiovascular events. Levels of creatinine, estimated glomerular filtration rate (eGFR, calculated by CKD-EPI formula), hemoglobin (Hb), albumin, C-reactive protein, parathyroid hormone (PTH), and vitamin D were evaluated as routine measurements before and after vaccination. Blood samples were taken for evaluation of anti-SARS-CoV-2 antibodies, lymphocyte subpopulations, and immunoglobulin (Ig)-G, M, A three to six weeks after vaccination. We continued follow-up for allograft rejection reactions after vaccination.

### 2.1. Measurement of SARS-CoV-2-Specific Antibodies

Anti-SARS-CoV-2 antibodies were evaluated using QuantiVac ELISA assay (Euroimmun), which provides quantitative in vitro determination of human antibodies of the IgG class against SARS-CoV-2 spike proteins in serum, according to the manufacturer‘s recommendations. Values were given in BAU/mL (BAU—binding antibody units). A result ≥35.2 BAU/mL was interpreted as a seropositive.

### 2.2. Evaluation of Lymphocyte Subpopulations

Quantification of lymphocyte subpopulations in blood was performed by flow cytometry (BD FACSLyric™, BD Biosciences, San Diego, CA, USA). After blood incubation with a monoclonal antibodies mix (BD Multitest™ 6-color TBNK reagent, BD Biosciences, San Diego, CA, USA) and erythrocytes lysis, prepared samples were acquired and analyzed on the BD FACSLyric system with BD FACSuite Clinical software (BD Biosciences, San Diego, CA, USA). During data analysis, the lymphocyte region was gated and the absolute numbers (cells/L) of lymphocyte subpopulations in the sample were determined. T, B and NK cells were characterized by expression of CD3, CD4, CD8, CD19 and CD16/56.

### 2.3. Statistical Methods

Statistical analysis was performed using SPSS 22.0 (Statistical Package for Social Science 22 for Windows). Continuous variables have been described as mean with standard deviation or median, according to data distribution. Parameter distribution was assessed using the Kolmogorov-Smirnov test. According to the level of antibodies against SARS-CoV-2 spike proteins, patients were divided into a seropositive group (anti-SARS-CoV-2 ≥ 35.2 BAU/mL), and a seronegative group (anti-SARS-CoV-2 < 35.2 BAU/mL). For comparison of seropositive and seronegative patients’ results, groups were analyzed by means of an independent t-test or the Mann-Whitney U test. Categorical variables have been described as absolute frequencies and percentages and analyzed by Fisher’s Exact test. Bivariate correlations of antibody titers with other variables were tested calculating Spearman’s coefficient. The significant variables that were found in univariate analysis were included into a binary logistic regression model for the risk of seronegativity. Changes in the variables before and after vaccination were assessed by paired t test and Wilcoxon signed-rank test for related samples. *p* < 0.05 was considered statistically significant for all analyses.

## 3. Results

The median age of studied patients was 55 (21–82) years: 88 (62.4%) men, 53 (37.6%) women.

Results of laboratory tests of kidney transplant patients before and after full vaccination against COVID-19 are summarized in Table 1.

Although there are statistically significant differences between eGFR, serum albumin before and after vaccination, these differences are not clinically significant. Differences in leucocyte and lymphocyte count obtained from SARS-CoV-2 seropositive and seronegative groups were studied in more detail.

Only 39 patients (29%) with kidney transplant developed an antibody response against SARS-CoV-2 (≥35.2 BAU/mL) after full vaccination against COVID-19.

Two groups of patients—with positive and negative antibody response—were compared according to the possible predictive factors (Table 2).

Out of all measures—creatinine level, eGFR, Hb before and after vaccination, and IgG after vaccination differed significantly between the groups. SARS-CoV-2 seropositive subjects with kidney transplants had higher levels of Hb, eGFR, IgG, and lower creatinine levels (Table 2). However, the amount of lymphocyte subsets (CD3+, CD4+, CD8+, CD19+, CD16+/56+, CD4/CD8) did not differ significantly between these groups (Table 3).

Comparison of SARS-CoV-2 antibody response in studied groups in relation to comorbid condition and concomitant medication is shown in Table 4. There were no statistically significant differences between the studied groups.

Statistically significant difference was noticed for a use of immunosuppression: antibody response to COVID-19 vaccine was better in patients, who were using sirolimus, and did not use mycophenolate mofetil (MMF) (Table 5).

Multivariate binary logistic regression analysis was performed, including age and most potent factors, tested after vaccination for evaluation of predictive factors for good antibody response after COVID-19 vaccination in kidney transplant patients (Table 6). Analysis showed that predictive factors for good response to COVID-19 vaccine are better kidney function, higher hemoglobin level, and no use of mycophenolate mofetil for immunosuppression.

Additional evaluation of kidney transplant patients with positive anti-SARS-CoV-2 antibodies after COVID-19 vaccination (separate analysis of 39 responders) showed that there is no correlation between anti-SARS-CoV-2 titer and patient’s age, level of IgA, M, G, vitamin D concentration, total leucocyte and lymphocyte count, MMF dose, and other routine laboratory test results before and after vaccination. However, there was a significant negative correlation between anti-SARS-CoV-2 antibody titer and CD4/CD8 ratio (Spearman’s correlation coefficient −0.4, *p* = 0.02) in responders. Anti-SARS-CoV-2 antibodies titer significantly correlated with percentage of CD8+ cells (r = 0.4, *p* = 0.01) and negatively correlated with percentage of CD19+ cells (r = −0.37, *p* = 0.02), but there was no significant correlation with absolute numbers of lymphocyte subpopulations.

Evaluation of leucocyte and lymphocyte count before and after COVID-19 vaccination showed an increase of total leucocyte count after vaccination in the total studied population, and in the group with a positive response by anti-SARS-CoV-2 antibody production (Table 7).

We also looked at the correlation of BNT162b2 vaccination and acute rejection. Out of 167 fully vaccinated patients, there were six (3.6%) biopsy proven rejection reactions during the nine months of follow-up (until November 2021): in one patient mixt (humoral and cellular) rejection diagnosed one month after second dose (this patient was known for non-compliance), in one patient cellular rejection diagnosed two months after second dose, and in another four patients rejection was diagnosed 6–9 months after second dose of vaccine. Out of 150 patients who were not vaccinated in our clinic, there were 6 rejection reactions (4%) during the same period. Therefore, there is no clear association between rejection reaction and vaccination.

## 4. Discussion

This study was conducted to evaluate the humoral and cellular immune responses induced by SARS-CoV-2 mRNA vaccine (Pfizer-BioNTech) in kidney transplant patients. Key findings of a study—measurable antibody response was detected only in one third of patients; level of antibodies was associated with a relative amount of CD8+ cells in antibody responders. Kidney function, level of anemia, mycophenolate mofetil use were important for antibody response.

First data about the immunological response to SARS-CoV-2 in solid organ transplant (SOT) patients were received from those who recovered from COVID-19. Both T- and B-cell responses against SARS-CoV-2 were detected in the blood around 1 week after the onset of COVID-19 symptoms. In one of the studies [22], the authors did not observe strong differences in the formation of polyfunctional and memory SARS-CoV-2-reactive T cell responses between transplanted and non-transplanted patients. Transplanted patients showed similar titers of neutralizing antibodies as compared to non-transplanted-patients. The authors presented the ability to generate SARS-CoV-2-specific immunity in immunosuppressed patients, raising hope for effective vaccination in this cohort [22]. These data were confirmed in another study performed by Favà and co-authors [23]: although in 28 SOT patients with moderate/severe COVID-19 there was a certain delay achieving immune responses, lower IgG seroconversion rates and cytokine-producing T-cell frequencies, but a similarly robust serological and functional T-cell immune response comparable to that of immunocompetent patients was detected during early convalescence [23].

However, the situation after vaccination appeared to be different. Investigation of early serological response after first dose of COVID-19 Pfizer/BioNTech (BNT162b2) mRNA vaccines in 74 kidney transplant recipients showed a positive antibody level in only three transplant recipients at day 36 [24]. In the study with 436 SOT patients, antibodies were detectable in 31 (41%), undetectable in 188 (53%) of kidney transplant patients after the first dose of the mRNA SARS-CoV-2 vaccine. Transplant recipients receiving anti–metabolite maintenance immunosuppression therapy were less likely to develop an antibody response than those not receiving such therapy (37% vs. 63%, respectively). Older transplant recipients were less likely to develop an antibody response. Those who received mRNA-1273 were more likely to develop an antibody response than those receiving BNT162b2 (69% vs. 31%, respectively, *p* = 0.003). These results contrast with the robust early immunogenicity observed in mRNA vaccine trials, including 100% anti-spike seroconversion by day 15 following vaccination with mRNA-12735 and by day 21 following vaccination with BNT162b2 [25].

In our study we did not evaluate quantitative antibody response after the first dose of COVID-19 vaccine. That is why we can compare our study data with the reports of immune response after the second dose only. Our data showed that only 29% of kidney transplant recipients become SARS-CoV-2 positive three to six weeks after the second dose of the BNT162b2 (Pfizer-BioNTech) vaccine. Such a low response to COVID-19 vaccination of kidney transplant patients was confirmed in few already published studies. In a German study only 4 of 39 and 1 of 39 transplanted individuals showed IgA and IgG seroconversion 8 and 23 days after vaccination. Although most transplanted patients mounted spike-specific T helper cell responses, frequencies were significantly reduced compared with those in controls, and this was accompanied by a broad impairment in effector cytokine production, memory differentiation, and activation-related signatures. Spike-specific CD8+ T cell responses were almost undetectable in transplant patients [26]. In another study, twenty-three renal transplant recipients of the Nierenzentrum Kronach, Germany were evaluated 2 weeks after standard protocol-based vaccination of two doses of the mRNA-based SARS-CoV-2 vaccine BNT162b2. Only 5 of the 23 (22%) renal transplant recipients tested positive for SARS-CoV-2 IgG antibodies after vaccination in contrast to 23 (100%) healthy controls (22% vs. 100%, *p* = 0.0001). In addition, the mean SARS-CoV-2 IgG titer of renal transplant recipients was significantly lower (50.9 +/− 138.7 vs. 727.7 +/− 151.3, *p* = 0.0001) [27].

The up to date published studies are small, and only some of them tried to find the most important factors associated to non-responsiveness. In a study with 45 kidney transplant recipients, only 17.8% of patients developed anti-spike SARSCoV-2 antibodies after two injections of mRNA BNT162b2 vaccine. In univariate analysis, predictive factors for a positive antibody response were the duration of kidney transplantation (*p* = 0.003) and a cyclosporine-based immunosuppressive regimen (*p* = 0.0003). T cell counts were not associated with the detection or the magnitude of the antibody but were associated with T cell response to the vaccine in univariate analysis (*p* = 0.01 for CD3, *p* = 0.05 for CD4, *p* = 0.03 for CD8) [28]. We did not confirm the association of antibody response with the duration of kidney transplantation and cyclosporine use. Of note, cyclosporine is used in our “older days” transplants, most of current kidney transplant patients receive tacrolimus, so cyclosporine use may correlate with longer duration of kidney transplantation also in other transplant centers. We confirmed no association between anti-spike SARSCoV-2 antibodies and T cell counts in our study.

The prospective study evaluated 117 SARS-CoV-2-naïve recipients of either kidney or kidney-pancreas grafts with assessment of IgM/IgG spike (S) antibodies and ELISpot against the nucleocapside (N) and the S protein at baseline and two weeks after receiving the second dose of the mRNA-1273 (Moderna) vaccine. Thirty-five patients (29.9%) developed either IgG or IgM two weeks after the second dose of the mRNA-1273 vaccine. At multivariable analysis, only baseline immunosuppression was significantly associated with no-response to the vaccine. 65.0% of patients developed either humoral or cellular response, while 35.0% did not develop any kind of response. Both humoral (either IgG or IgM) and cellular response (S-ELISpot positivity) was observed in 23 patients (19.6%). Considering vaccine non-responders (both IgG/IgM and S(ELISPot)-negative), the factors that were associated with an absence of response at univariable analysis were diabetes (OR [95%CI] 3.41 [1.41–8.22], *p* = 0.006), receiving ATG during the last year (OR [95%CI] 10.07 [2.64–38.31], *p* = 0.001), lymphopenia (OR [95%CI] 3.82 [1.64–8.89], *p* = 0.001), time from transplant <1 year (OR [95%CI] 3.51 [1.52–8.08], *p* = 0.003) and eGFR < 30 mL/min/1.73 m^2^ (OR [95%CI] 4.95 [1.48–16.46], *p* = 0.009). At multivariable analysis, diabetes and treatment with anti-thymocytes globulins during the last year were associated with vaccine no-response [29]. Association of diabetes with non-responsiveness to vaccination against COVID-19 was not confirmed in other studies nor in our patients. We did not collect data about ATG use in the year before vaccination.

More similar results with our study were reported from an Israeli group. They analyzed the humoral response following full vaccination with the BNT162b2 (Pfizer-BioNTech) in 136 kidney transplant recipients and compared it to 25 controls. All participants in the control group had a positive antibody response to spike protein, while only 51 of 136 transplant recipients (37.5%) had positive serology (*p* < 0.001). Participants with a positive anti-spike serology were significantly younger, had a shorter period of time on maintenance dialysis before transplantation, and had a higher prevalence of living donors. The main difference in immunosuppression between the two groups was a lower rate of treatment with MMF and a lower rate of triple maintenance immunosuppression in responders. Seropositive patients had a significantly higher eGFR, and higher mean hemoglobin and lymphocyte counts. A longer period of time since transplantation was significantly associated with positive response to the vaccination. When multivariate analysis was performed, variables associated with negative humoral response were: older age (odds ratio 1.66 [95% confidence interval 1.17–2.69], *p* = 0.026), high-dose corticosteroids in the last 12 months (1.3 [1.09–1.86], *p* = 0.048], maintenance with triple immunosuppressive medications (1.43 [1.06–2.15], *p* = 0.038], and a regimen that includes MMF (1.47 [1.26–2.27], *p* = 0.049] [30]. Our data confirm a similar response to the vaccination rate. We did not find an association with age, but we could confirm lower GFR and lower hemoglobin as important factors for non-responsiveness in multifactorial analysis. As for immunosuppression, only MMF use was associated with non-responsiveness in multifactorial analysis. A worse response after vaccination when using MMF was confirmed with other vaccines in kidney transplant patients. In one of the studies, the response rate after influenza vaccination decreased in a dose-dependent manner in patients receiving mycophenolate mofetil (MMF), while seroprotection was comparable to non-MMF users. This implies that response to vaccines may be appropriate, but the quality of immune response may be impaired and likely depends on the dose of MMF used [15]. We did not find a correlation of MMF dose with antibody titers in BNT162b2 (Pfizer-BioNTech) responders.

The association of worse kidney function and mycophenolate mofetil use with non-responsiveness to vaccination was also observed in a study of 308 kidney transplant recipients. Only 112 (36.4%) tested positive for anti-S antibodies two to four weeks after receiving the second dose of the BNT162b2 vaccine. Factors associated with antibody response were higher eGFR (OR 1.025 per mL/min/1.73 m^2^, 95% CI 1.014–1.037, *p* < 0.001), lower mycophenolic acid dose (OR 2.347 per 360 mg decrease, 95%CI 1.782–3.089, *p* < 0.001), younger age (OR 1.032 per year decrease, 95%CI 1.015–1.05, *p* < 0.001) and lower calcineurin inhibitor (CNI) blood level (OR 1.987, 95%CI 1.146–3.443, *p* 0.014) [31].

A highest rate of response after full vaccination of kidney transplant patients until now is reported in a study from France. They published the follow-up data of 205 kidney transplant patients who received two doses of the mRNA-1273 SARS-CoV-2 vaccine. 48% of study patients responded to vaccination. Patients who received their first transplant had a longer duration from transplant, experienced better graft function, and experienced lower levels of overall immunosuppression mounted a stronger immune response. Patients treated with calcineurin inhibitors, mycophenolate mofetil, or steroids showed significantly lower anti–SARS-CoV-2 antibody titers [32].

Lymphocytopenia and decreased CD3, CD4, and CD8 counts were common in patients with COVID-19 and correlate with disease severity [33]. Severe peripheral lymphopenia, especially a decrease of T cells observed during COVID-19, was also confirmed in other studies convalescence [23,34,35,36]. However, in the study analyzing patients with a positive response by antibody production after COVID-19 vaccination, significant increase of lymphocyte count was observed [30]; other studies (including our study) did not have similar findings [24]. In kidney transplant patients, T cell counts were not associated with the magnitude of the antibody, but were associated with T cell response to the vaccine in univariate analysis (*p* = 0.01 for CD3, *p* = 0.05 for CD4, *p* = 0.03 for CD8) [28].

During clinical evaluation of lymphocyte subpopulations by immunophenotyping, the absolute number of cells are mostly used to assess immunosuppression or immunodeficiencies, but in our study we did not find a statistically significant difference between anti-SARS-CoV-2 seropositive and seronegative patient groups with kidney transplant. The relative number of lymphocyte subpopulations can be useful to determine effects induced by viral infection, especially in immunocompromised patients; relative changes of CD8+ and CD4+ cells are reflected on CD4/CD8 ratio and worsen prognosis, while the difference in absolute numbers of lymphocyte subpopulations may not be detected. Similar mechanisms of action of vaccines could be expected if specific T cell immunity is achieved, depending on vaccine type [36,37,38].

In our study, there was no correlation between anti-SARS-CoV-2 antibodies and the absolute number of lymphocytes or their subpopulations (T, B, NK cells). However, there was a significant negative correlation between anti-SARS-CoV-2 antibody level and CD4/CD8 ratio (Spearman’s correlation coefficient −0.4, *p* = 0.02), and the relative number of CD8+ T cells (r = 0.41, *p* = 0.01) in responders to the COVID-19 vaccine. It could be explained by the recent finding from Rha et al. [39] during analysis of phenotypes of SARS-CoV-2-specific T cells elicited by infection or vaccination. They showed that SARS-CoV-2-specific CD8+ T cells are not exhausted, but functional. Furthermore, COVID-19 vaccines induce SARS-CoV-2-specific T-cell responses by forming activated and memory CD8+ T cells as described by Teijaro, J.R. and others [40,41]. In our study, anti-SARS-CoV-2 antibody titers did not correlate to CD19+ absolute cell count, but there was a significant negative correlation with percentage of CD19+ cells (r = −0.37, *p* = 0.02). Changes in the proportion of lymphocyte subpopulations after vaccination could be explained by SARS-CoV-2-specific CD8+ T cells formation and might be related to B cell activity or their phenotypes. It can be hypothesized that due to enhanced chemotaxis, part of B cells migrate to the site of vaccination and other lymphoid tissues, where they are activated and transformed to plasma cells producing specific immunoglobulins; in such a way the proportion of B lymphocytes among other lymphocytes in peripheral blood may be reduced, but changes in absolute numbers of CD19+ cells are not significant enough to be detected [40,42].

In contrast, another study showed that anti-SARS-CoV-2 antibody titers and B cell proportions after Rituximab treatment (B lymphocyte depletion) directly correlate, and for seroconversion only a small amount of B lymphocytes (<1%) is needed [43]. Therefore, better understanding of SARS-CoV-2-specific T cell activation and mediation is required.

In summary, the current vaccination strategy for kidney transplant recipients may not provide effective protection against COVID-19. Individual vaccination strategies might be needed and evaluated in clinical trials in the immunosuppressive cohort. In the meantime, these studies reinforce the need to continue good public health measures, such as hand hygiene, mask wearing, and social distancing, as practical means to help limit the spread of COVID-19 in our renal communities. In light of the reduced response in patients who received a kidney transplant, strategies that promote vaccination of close household contacts to provide so-called “ring vaccination” of those closest to transplant recipients seem a logical approach to reduce the chance of direct household spread [44].

## 5. Conclusions

According to the levels of anti-SARS-CoV-2 antibodies, only one third of patients with kidney transplants develop sufficient antibody response after full COVID-19 vaccination with Pfizer-BioNTech. Factors that may significantly influence the adequacy of response to the COVID-19 vaccine in these patients are: better kidney function, higher hemoglobin levels, and no use of mycophenolate mofetil for immunosuppression.

Even though in kidney transplant patients the level of humoral response developed after COVID-19 vaccination with Pfizer-BioNTech was not related to the absolute number of different lymphocyte subpopulations, it correlates with the relative number of CD8+ cells and negatively with the CD4/CD8 ratio in responders. This fact let us hypothesize about the importance of the relation between cellular and humoral immunity as well as other factors in the development of specific responses after COVID-19 vaccination in kidney transplant patients and emphasized the necessity of further extended studies.

## Figures and Tables

**Table 1:** Results of laboratory tests of kidney transplant patients before and after full vaccination against COVID-19.

Laboratory Test	Before Vaccination Mean (Standard Deviation)	After Vaccination Mean (Standard Deviation)	*p*
eGFR * (CKD-EPI, mL/min/1.73 m^2^)	53.2 (s = 20)	51.3 (s = 21)	0.007
C-reactive protein (mg/L)	5.3 (s = 1.3)	5.7 (s = 3.3)	0.1
Serum albumin (g/L)	38 (s = 4.7)	41 (s = 3.3)	<0.001
Hemoglobin (mg/L)	135 (s = 15.4)	136 (s = 14)	0.07
Leucocyte count (×10^9^/L)	7.3 (s = 2.2)	8.3 (s = 5.2)	0.02
Lymphocyte count (×10^9^/L)	2.0 (s = 2.7)	2.1 (2.2)	0.6
Vitamin D level (nmol/L)	62.5 (s = 26.6)	66.7 (s = 28.3)	0.06

*—estimated glomerular filtration rate (eGFR).

**Table 2:** Comparison of evaluated demographic and routine laboratory results in SARS-CoV-2 seropositive and seronegative groups with kidney transplant.

	SARS-CoV-2 Seronegative (*n* = 97)		SARS-CoV-2 Seropositive (*n* = 39)		*p*
	Mean (s)	Median (Min–Max)	Mean (s)	Median (Min–Max)	
Age, years	53 (13)	55 (25–74)	52 (14)	55 (21–82)	0.6
Years after transplantation	6.1 (4.6)	5.5 (0.3–20.7)	7.4 (5.1)	5.8 (0.9–21.8)	0.2
Creatinine before vaccination (mcmol/L)	145 (63)	123 (68–446)	116 (45)	110 (51–246)	0.004
eGFR * before vaccination (mL/min/1.73 m^2^)	49 (18)	50 (15–102)	64 (22)	59 (23–117)	<0.001
Hemoglobin before vaccination (g/L)	132 (15)	132 (84–185)	142 (14)	141 (114–176)	<0.001
Creatinine after vaccination (mcmol/L)	150 (66)	128 (68–435)	121 (48)	113 (22–118)	0.012
eGFR * after vaccination (mL/min/1.73 m^2)^	47 (18)	46 (15–96)	62 (24)	61 (22–118)	<0.001
Hemoglobin after vaccination (g/L)	134 (14)	134 (101–169)	142 (14)	141 (119–173)	0.004
Immunoglobulin G (g/L)	8.4 (2.9)	8.4 (0.3–17)	9.6 (3.1)	9.8 (0.8–17)	0.035

*—estimated glomerular filtration rate (eGFR).

**Table 3:** Comparison of lymphocyte subpopulations in SARS-CoV-2 seropositive and seronegative groups with kidney transplant after COVID-19 vaccination.

Lymphocyte Subpopulations	SARS-CoV-2 Seronegative (*n* = 97)	SARS-CoV-2 Seropositive (*n* = 39)	*p*
T lymphocytes—CD3 (×10^9^/L)	81 (s = 7.8)	81 (s = 9.3)	0.7
T helpers—CD4+ (×10^9^/L)	0.8 (s = 0.4)	0.7(s = 0.3)	0.4
T supressors—CD8+ (×10^9^/L)	0.6 (s = 0.3)	0.6 (s = 0.3)	0.98
B lymphocytes—CD19+ (×10^9^/L)	0.1 (s = 0.07)	0.1 (s = 0.1)	0.9
Natural killers CD16+/56+ (×10^9^/L)	0.2 (s = 0.14)	0.2 (s = 0.15)	0.4
CD4/CD8 ratio	1.5 (s = 0.7)	1.3 (s = 0.5)	0.7

**Table 4:** Comparison of comorbid condition and concomitant medication in SARS-CoV-2 seropositive and seronegative groups.

	SARS-CoV-2 Seronegative (*n* = 97)	SARS-CoV-2 Seropositive (*n* = 39)	*p*
Men	60 (62%)	24 (62%)	0.97
Women	37 (38%)	15 (39%)	0.97
Diabetes	18 (19%)	5 (13%)	0.4
Cardiovascular disease	23 (24%)	10 (26%)	0.8
Oncological disease	3 (3%)	4 (10%)	0.09
Use of ACEi	43 (44%)	15 (39%)	0.5
Use of ARB	26 (27%)	11 (28%)	0.9
Use of NSAID	2 (2%)	2 (5%)	0.3
Use of PPI	61 (63%)	23 (59%)	0.7
Use of statins	27 (59%)	28 (72%)	0.2
Use of aspirin	12 (12%)	6 (15%)	0.6

**Table 5:** Comparison of immunosuppressants in SARS-CoV-2 seropositive and seronegative groups.

Immunosuppressive Medications	SARS-CoV-2 Seronegative (*n* = 97)	SARS-CoV-2 Seropositive (*n* = 39)	*p*
Use of steroids	84 (87%)	36 (92%)	0.6
Use of mycophenolate mofetil	93 (96%)	20 (51%)	<0.001
Use of CNIs *	90 (93%)	33 (85%)	0.14
Use of cyclosporine	36 (37%)	11(29%)	0.4
Use of tacrolimus	59 (61%)	21 (55%)	0.6
Use of sirolimus	5 (5%)	8 (21%)	0.01

*—calcineurin inhibitor (CNI).

**Table 6:** Multivariate logistic regression analysis model for the risk of SARS-CoV-2 seronegativity after COVID-19 vaccination in kidney transplant patients.

Factor	B	*p*	Exp (B)	95% CI
Age	−0.008	0.7	0.992	0.954–1.03
eGFR * after vaccination	0.033	0.011	1.034	1.008–1.060
Hemoglobin after vaccination	0.05	0.011	1.05	1.012–1.095
Immunoglobulin G concentration	0.117	0.163	1.12	0.953–1.326
Use of mycophenolate mofetil	3.9	<0.001	51	10–251
Use of Sirolimus	−0.013	0.989	0.99	0.15–6.4
Constant	−11,19	<0.001	0.000	

*—estimated glomerular filtration rate (eGFR).

**Table 7:** Change of leucocyte and lymphocyte count before and after COVID-19 vaccination in kidney transplant patients.

		Before Vaccination Mean (SD)	After Vaccination Mean (SD)	*p*
Total population	Leucocytes (×10^9^/L)	7.3 (2.2)	8.3 (5.2)	0.02
	Lymphocytes (×10^9^/L)	2.2 (2.7)	2.1 (2.2)	0.6
Non-responders (Anti-SARS-CoV-2 <35.2BAU/mL *)	Leucocytes (×10^9^/L)	7.3 (2.2)	8.4 (6.1)	0.1
	Lymphocytes (×10^9^/L)	1.7 (0.6)	1.9 (1.5)	0.2
Responders (Anti-SARS-CoV-2 ≥35.2 BAU/mL *)	Leucocytes (×10^9^/L)	7.0 (2.2)	8.0 (2.5)	0.001
	Lymphocytes (×10^9^/L)	2.6 (5.0)	2.4 (3.1)	0.8

*—binding antibody units (BAU).

## Data Availability

Not applicable.

## References

[1] Williamson E.J., Walker A.J., Bhaskaran K., Bacon S., Bates C., Morton C.E., Curtis H.J., Mehrkar A., Evans D., Inglesby P. (2020). Factors associated with COVID-19-related death using OpenSAFELY. Nature.

[2] Vanholder R. How Do Health Systems Meet the Challenge of Managing Chronic Diseases during COVID-19 and Beyond? Friends of Europe. https://www.friendsofeurope.org/insights/how-do-healthsystems-meet-the-challenge-of-managing-chronic-diseasesduring-covid-19-and-beyond/.

[3] Anonymous European Kidney Health Alliance (EKHA) Improve CKD Prevention, Treatment and Care in the Aftermath of COVID-19. http://ekha.eu/blog/ekha-launches-a-call-to-action-to-improve-ckd-prevention-treatment-and-care-in-the-aftermath-of-covid-19/.

[4] ERA-EDTA Council (2021). ERACODA Working Group Chronic kidney disease is a key risk factor for severe COVID-19: A call to action by the ERA-EDTA. Nephrol. Dial. Transplant..

[5] Ruan Q., Yang K., Wang W., Jiang L., Song J. (2020). Correction to: Clinical predictors of mortality due to COVID-19 based on an analysis of data of 150 patients from Wuhan, China. Intensive Care Med..

[6] Zhou F., Yu T., Du R., Fan G., Liu Y., Liu Z., Xiang J., Wang Y., Song B., Gu X. (2020). Clinical course and risk factors for mortality of adult inpatients with COVID-19 in Wuhan, China: A retrospective cohort study. Lancet.

[7] Banerjee D., Popoola J., Shah S., Ster I.C., Quan V., Phanish M. (2020). COVID-19 infection in kidney transplant recipients. Kidney Int..

[8] Coates P.T., Wong G., Drueke T., Rovin B., Ronco P. (2020). Associate Editors, for the Entire Editorial Team Early experience with COVID-19 in kidney transplantation. Kidney Int..

[9] Fernández-Ruiz M., Andrés A., Loinaz C., Delgado J.F., López-Medrano F., San Juan R., González E., Polanco N., Folgueira M.D., Lalueza A. (2020). COVID-19 in solid organ transplant recipients: A single-center case series from Spain. Am. J. Transplant..

[10] Elias M., Pievani D., Randoux C., Louis K., Denis B., Delion A., Le Goff O., Antoine C., Greze C., Pillebout E. (2020). COVID-19 Infection in Kidney Transplant Recipients: Disease Incidence and Clinical Outcomes. J. Am. Soc. Nephrol..

[11] Combe C., Kirsch A.H., Alfano G., Luyckx V.A., Shroff R., Kanbay M., van der Sande F., Basile C. (2021). EUDIAL Working Group of the ERA-EDTA At least 156 reasons to prioritize COVID-19 vaccination in patients receiving in-centre haemodialysis. Nephrol. Dial. Transplant..

[12] (2021). Updated Joint AST/ASTS/ISHLT Statement about Vaccine Efficacy in Organ Transplant Recipients. https://www.myast.org/updated-joint-astastsishlt-statement-about-vaccine-efficacy-organ-transplant-recipients.

[13] Centers for Disease Control and Prevention COVID-19 Vaccination Program Operational Guidance. https://www.cdc.gov/vaccines/covid-19/covid19-vaccination-guidance.htm.

[14] ECD COVID-19 Vaccination and Prioritisation Strategies in the EU/EEA. https://www.ecdc.europa.eu/sites/default/files/documents/COVID-19-vaccination-and-prioritisation-strategies.pdf.

[15] Kronbichler A., Anders H.J., Fernandez-Juárez G.M., Floege J., Goumenos D., Segelmark M., Tesar V., Turkmen K., van Kooten C., Bruchfeld A. (2021). Immunonephrology Working Group of the ERA-EDTA (European Renal Association—European Dialysis, Transplant Association) Recommendations for the use of COVID-19 vaccines in patients with immune-mediated kidney diseases. Nephrol. Dial. Transplant..

[16] Thanachartwet V., Phumratanaprapin W., Desakorn V., Sahassananda D., Wattanagoon Y., Chaiprasert A., Aimpun P., Supaporn T. (2007). Viral hepatitis infections among dialysis patients: Thailand registry report. Nephrology (Carlton).

[17] Ramezani A., Eslamifar A., Banifazl M., Ahmadi F., Maziar S., Razeghi E., Kalantar E., Amirkhani A., Aghakhani A. (2009). Efficacy and long-term immunogenicity of hepatitis B vaccine in haemodialysis patients. Int. J. Clin. Pract..

[18] DaRoza G., Loewen A., Djurdjev O., Love J., Kempston C., Burnett S., Kiaii M., Taylor P.A., Levin A. (2003). Stage of chronic kidney disease predicts seroconversion after hepatitis B immunization: Earlier is better. Am. J. Kidney Dis..

[19] Sanchez-Fructuoso A.I., Prats D., Naranjo P., Fernández-Pérez C., González M.J., Mariano A., González J., Figueredo M.A., Martin J.M., Paniagua V. (2000). Influenza virus immunization effectivity in kidney transplant patients subjected to two different triple-drug therapy immunosuppression protocols: Mycophenolate versus azathioprine. Transplantation.

[20] Cavdar C., Sayan M., Sifil A., Artuk C., Yilmaz N., Bahar H., Camsari T. (2003). The comparison of antibody response to influenza vaccination in continuous ambulatory peritoneal dialysis, hemodialysis and renal transplantation patients. Scand. J. Urol. Nephrol..

[21] Vilchez R.A., McCurry K., Dauber J., Lacono A., Griffith B., Fung J., Kusne S. (2002). Influenza virus infection in adult solid organ transplant recipients. Am. J. Transplant..

[22] Thieme C.J., Anft M., Paniskaki K., Blazquez-Navarro A., Doevelaar A., Seibert F.S., Hoelzer B., Konik M.J., Meister T.L., Pfaender S. (2021). The Magnitude and Functionality of SARS-CoV-2 Reactive Cellular and Humoral Immunity in Transplant Population Is Similar to the General Population Despite Immunosuppression. Transplantation.

[23] Favà A., Donadeu L., Sabé N., Pernin V., González-Costello J., Lladó L., Meneghini M., Charmetant X., García-Romero E., Cachero A. (2021). SARS-CoV-2-specific serological and functional T cell immune responses during acute and early COVID-19 convalescence in solid organ transplant patients. Am. J. Transplant..

[24] Danthu C., Hantz S., Dahlem A., Duval M., Ba B., Guibbert M., El Ouafi Z., Ponsard S., Berrahal I., Achard J.M. (2021). Humoral Response after SARS-CoV-2 mRNA Vaccination in a Cohort of Hemodialysis Patients and Kidney Transplant Recipients. J. Am. Soc. Nephrol..

[25] Boyarsky B.J., Werbel W.A., Avery R.K., Tobian A.A.R., Massie A.B., Segev D.L., Garonzik-Wang J.M. (2021). Immunogenicity of a Single Dose of SARS-CoV-2 Messenger RNA Vaccine in Solid Organ Transplant Recipients. JAMA.

[26] Sattler A., Schrezenmeier E., Weber U.A., Potekhin A., Bachmann F., Straub-Hohenbleicher H., Budde K., Storz E., Proß V., Bergmann Y. (2021). Impaired humoral and cellular immunity after SARS-CoV2 BNT162b2 (Tozinameran) prime-boost vaccination in kidney transplant recipients. J. Clin. Investig..

[27] Korth J., Jahn M., Dorsch O., Anastasiou O.E., Sorge-Hädicke B., Eisenberger U., Gäckler A., Dittmer U., Witzke O., Wilde B. (2021). Impaired Humoral Response in Renal Transplant Recipients to SARS-CoV-2 Vaccination with BNT162b2 (Pfizer-BioNTech). Viruses.

[28] Bertrand D., Hamzaoui M., Lemée V., Lamulle J., Hanoy M., Laurent C., Lebourg L., Etienne I., Lemoine M., Le Roy F. (2021). Antibody and T Cell Response to SARS-CoV-2 Messenger RNA BNT162b2 Vaccine in Kidney Transplant Recipients and Hemodialysis Patients. J. Am. Soc. Nephrol..

[29] Cucchiari D., Egri N., Bodro M., Herrera S., Del Risco-Zevallos J., Casals-Urquiza J., Cofan F., Moreno A., Rovira J., Banon-Maneus E. (2021). Cellular and humoral response after mRNA-1273 SARS-CoV-2 vaccine in kidney transplant recipients. Am. J. Transplant..

[30] Grupper A., Katchman H. (2021). Reduced humoral response to mRNA SARS-CoV-2 BNT162b2 vaccine in kidney transplant recipients without prior exposure to the virus: Not alarming, but should be taken gravely. Am. J. Transplant..

[31] Rozen-Zvi B., Yahav D., Agur T., Zingerman B., Ben-Zvi H., Atamna A., Tau N., Mashraki T., Nesher E., Rahamimov R. (2021). Antibody response to SARS-CoV-2 mRNA vaccine among kidney transplant recipients: A prospective cohort study. Clin. Microbiol. Infect..

[32] Benotmane I., Gautier-Vargas G., Cognard N., Olagne J., Heibel F., Braun-Parvez L., Martzloff J., Perrin P., Moulin B., Fafi-Kremer S. (2021). Low immunization rates among kidney transplant recipients who received 2 doses of the mRNA-1273 SARS-CoV-2 vaccine. Kidney Int..

[33] Azzi Y., Bartash R., Scalea J., Loarte-Campos P., Akalin E. (2021). COVID-19 and Solid Organ Transplantation: A Review Article. Transplantation.

[34] Du R.H., Liang L.R., Yang C.Q., Wang W., Cao T.Z., Li M., Guo G.Y., Du J., Zheng C.L., Zhu Q. (2020). Predictors of mortality for patients with COVID-19 pneumonia caused by SARS-CoV-2: A prospective cohort study. Eur. Respir. J..

[35] Xu B., Fan C.Y., Wang A.L., Zou Y.L., Yu Y.H., He C., Xia W.G., Zhang J.X., Miao Q. (2020). Suppressed T cell-mediated immunity in patients with COVID-19: A clinical retrospective study in Wuhan, China. J. Infect..

[36] Ganji A., Farahani I., Khansarinejad B., Ghazavi A., Mosayebi G. (2020). Increased expression of CD8 marker on T-cells in COVID-19 patients. Blood Cells Mol. Dis..

[37] Zielinski M., Tarasewicz A., Zielinska H., Jankowska M., Moszkowska G., Debska-Slizien A., Rutkowski B., Trzonkowski P. (2017). Impact of donor and recipient human cytomegalovirus status on kidney transplantation. Int. Immunol..

[38] Claire-Anne S., Plotkin S., Orenstein W., Paul A., Offit P., Edward K. (2018). Vaccine Immunology. Plotkin’s Vaccine.

[39] Rha M.S., Jeong H.W., Ko J.H., Choi S.J., Seo I.H., Lee J.S., Sa M., Kim A.R., Joo E.J., Ahn J.Y. (2021). PD-1-Expressing SARS-CoV-2-Specific CD8(+) T Cells Are Not Exhausted, but Functional in Patients with COVID-19. Immunity.

[40] Teijaro J.R., Farber D.L. (2021). COVID-19 vaccines: Modes of immune activation and future challenges. Nat. Rev. Immunol..

[41] Jung M.K., Shin E.C. (2021). Phenotypes and Functions of SARS-CoV-2-Reactive T Cells. Mol. Cells.

[42] Palm A.E., Henry C. (2019). Remembrance of Things Past: Long-Term B Cell Memory After Infection and Vaccination. Front. Immunol..

[43] Mrak D., Tobudic S., Koblischke M., Graninger M., Radner H., Sieghart D., Hofer P., Perkmann T., Haslacher H., Thalhammer R. (2021). SARS-CoV-2 vaccination in rituximab-treated patients: B cells promote humoral immune responses in the presence of T-cell-mediated immunity. Ann. Rheum. Dis..

[44] Ikizler T.A., Coates P.T., Rovin B.H., Ronco P. (2021). Immune response to SARS-CoV-2 infection and vaccination in patients receiving kidney replacement therapy. Kidney Int..

